# Negative Interference by Rheumatoid Factor of Plasma B-Type Natriuretic Peptide in Chemiluminescent Microparticle Immunoassays

**DOI:** 10.1371/journal.pone.0105304

**Published:** 2014-08-21

**Authors:** Wen Fan, Lei Xu, Liangcai Xie, Decai Yang, Xuezheng Liu, Jiajun Zhang, Yirong Li, Cunjian Yi

**Affiliations:** 1 Department of Laboratory Medicine, Jingzhou First People's Hospital, Jingzhou, Hubei, China; 2 Department of Laboratory Medicine, Union Hospital, Tongji Medical College, Huazhong University of Science and Technology, Wuhan, Hubei, China; 3 Department of Obstetrics and Gynecology, Jingzhou First People's Hospital, Jingzhou, Hubei, China; Naval Research Laboratory, United States of America

## Abstract

**Background:**

The chemiluminescent microparticle immunoassay (CMIA) is widely used for the quantitative determination of B-type natriuretic peptide (BNP) in human ethylenediaminetetraacetic acid plasma. Rheumatoid factor (RF) is usually thought to result in a positive interference in immunoassays, but it is not clear whether its presence in plasma can lead to interferences in the CMIA of BNP.

**Methods:**

The estimation of BNP recovery was carried out by diluting high-concentration BNP samples with RF-positive or RF-negative plasma at a ratio of 1∶9. The diluted samples were then tested using the ARCHITECT i2000 System and ARCHITECT BNP Reagent Kits and the recovery was then calculated.

**Results:**

When the RF level ranged from 48 to 1420 IU/mL, the average recovery of BNP was 79.29% and 91.61% in the RF-positive and RF-negative plasma samples, respectively, and was thus significantly lower in the group of RF-positive plasma samples than in the group of RF-negative plasma samples. At a dilution of 1∶16, the measured BNP level increased by >36% in six of the seven RF-positive plasma samples. The recovery of BNP increased significantly in the RF-positive plasma samples after pretreatment with IgG-sensitive latex particles. In addition, The BNP recovery was not significantly related to the plasma RF at concentrations ranging from 48 to 2720 IU/mL.

**Conclusions:**

Measurement of BNP by CMIA is susceptible to interference from RF leading to predominantly (but not exclusively) lower results. Pretreatment of samples with blocking reagents is advisable prior to the initiation of denying patient's necessary treatment.

## Introduction

The rheumatoid factor (RF), a type of autoantibody against the fragment crystallizable portion of immunoglobulin (Ig) G, includes five subclasses of Ig, IgA, IgG, IgM, IgE, and IgD [1]–[2] found in 20% of serum/plasma of people over 60 years of age. RF is known to cause interference in immunoassays. In the two-site immunometric assay, there is an increased likelihood that RF forms a bridge between the capture antibody and assay antibody, which falsely increases the analyte concentrations [3]–[4]. Immunoassays that use either polyclonal or monoclonal antibodies can be affected. The presence of RF in the serum or plasma has been found to result in positive interferences in enzyme-linked immunosorbent assays (ELISAs) for hepatitis B virus surface antigen (HBsAg) [5]–[6], troponin immunoassays [7], thyroid function tests [8], tumor marker immunoassays [9], and cytokine immunoassays [10].

B-type natriuretic peptide (BNP), a member of the family of natriuretic peptides that were initially isolated from porcine brain tissue, is mostly synthesized and released into the blood in response to volume overload or conditions that cause ventricular stretch [11]–[12]. BNP is cleared from the circulation with a half-life of approximately 23 min. Levels of BNP have been shown to be elevated in patients with cardiac dysfunction. Plasma BNP levels provide clinically useful information concerning the diagnosis and management of left ventricular dysfunction and heart failure, which complements other diagnostic testing procedures (e.g., electrocardiograms, chest x-rays, and echocardiograms) [13]–[14]. In addition, BNP levels can be used to assess the severity of heart failure, as demonstrated by their correlation with New York Heart Association classifications [15]. The European Society of Cardiology has also included the use of BNP testing in their guidelines for the diagnosis of or to rule out heart failure [16].

The chemiluminescent microparticle immunoassay (CMIA) is widely used for the quantitative determination of BNP in human ethylenediaminetetraacetic acid (EDTA) plasma. Investigations by the National Center for Clinical Laboratories showed that most clinical laboratories used CMIA to determine plasma BNP in 2013 (www.clinet.com.cn), and that most of the clinical laboratories used the ARCHITECT BNP Reagent Kits (Abbott Laboratories, IL, USA). Multiple substances, including triglycerides and heparin, were thought to be potential sources of interference in the ARCHITECT BNP assay. However, it was not clear whether the presences of RF in plasma resulted in interferences in BNP CMIA. Generally, RF has been shown to cause a positive interference in immunoassay. A multicenter survey showed that about 8.7% of the 3445 immunoassay results from assays of 74 analytes in 10 donors,who suffering from several illnesses known to be associated with the presence of RF in their serum, were considered to be “false positive”[17]. But it was neglected that results of plasma myoglobin and hCG assays also increased after pretreatment with heterophil-blocking reagent at least [17], indicating that RF also caused false-negative results. Our group recently found that RF led to both negative and positive interferences in the serum HBsAg ELISA [5]–[6]. The negative interference had not drawn much attention until we found it in HBsAg ELISA, and it was still unclear whether the negative interference caused by RF was an anomaly produced by the HBsAg ELISA or was a denominator of immunoassays. In this study, we determined whether RF causes negative interference in plasma BNP CMIA.

## Materials and Methods

### Plasma samples

EDTA anti-coagulated whole blood samples were collected in the Jingzhou First People's Hospital and the Union Hospital Affiliated with Tongji Medical College, Huazhong University of Science and Technology in 2013. The plasma was isolated by centrifugation for 10 min at 3,400 revolutions per minute (rpm) and stored at −20°C. Fifty-seven RF-positive (RF≥20 IU/mL) plasma samples from patients with RA, 22 RF-negative (RF<20 IU/mL) plasma samples from healthy volunteers and 9 plasma samples with BNP levels ranging from 887 to 2230 pg/mL were collected. The study was approved by the Ethics Committee of the Jingzhou First People's Hospital and the Union Hospital Affiliated with Tongji Medical College, Huazhong University of Science and Technology. All of the patients signed an informed consent form.

### Determination of the plasma RF levels

The plasma RF levels were determined using the BNII System and N Latex RF Kit (Siemens Healthcare Diagnostics Inc., Newark, USA) according to the instruction manual.

### Quantitative determination of plasma BNP

Plasma BNP was determined by CMIA using the ARCHITECT i2000 System and ARCHITECT BNP Reagent Kits (Abbott Laboratories, IL, USA) as follows. Plasma and anti-BNP-coated paramagnetic microparticles were combined, and the BNP present in the plasma bound to the anti-BNP-coated microparticles. After washing, anti-BNP acridinium-labeled conjugate was added to create a reaction mixture. Following another wash cycle, pre-trigger and trigger solutions were added to the reaction mixture. The resulting chemiluminescent reaction was measured as relative light units by the ARCHITECT i2000 System optics. A direct relationship existed between the amount of BNP in the plasma and the relative light units.

### Estimation of BNP recovery

The estimation of BNP recovery was carried out by diluting high-concentration BNP with RF-positive or RF-negative plasma at a ratio of 1∶9, respectively. The diluted samples were then tested using the ARCHITECT i2000 System and ARCHITECT BNP Reagent Kits, and the BNP recovery was calculated as described in [18].

### Pretreating plasma samples with human IgG-sensitive latex particles

RF-positive plasma samples were mixed with human IgG-sensitive latex particles (Jiemen Biological and Technical Co., Shanghai, China) at a ratio of 1∶1. The mixtures were incubated at 37°C in a water bath for 1 h and then centrifuged at 13,000 rpm for 10 min. The supernatant was collected for the determination of the RF concentrations and BNP levels. A control group was included by blending RF-positive plasma with a buffer with no human IgG-sensitive latex particles (Jiemen Biological and Technical Co., Shanghai, China) at the same ratio.

### Dilution of high-concentration RF

RF-positive plasma samples were diluted 1∶2, 1∶4, 1∶8, and 1∶16 with the sample buffer in the ARCHITECT BNP Reagent Kits for the subsequent determination of BNP.

### Statistical analysis

Statistical analysis was performed using the SPSS 17.0 software package. Student's t test was used to compare the continuous data. The bivariate correlations test was used to analyze the correlation between the RF concentration and BNP recovery. A p value≤0.05 was considered to be statistically significant.

## Results

### BNP recovery in RF-positive plasma samples

**Figure 1:**
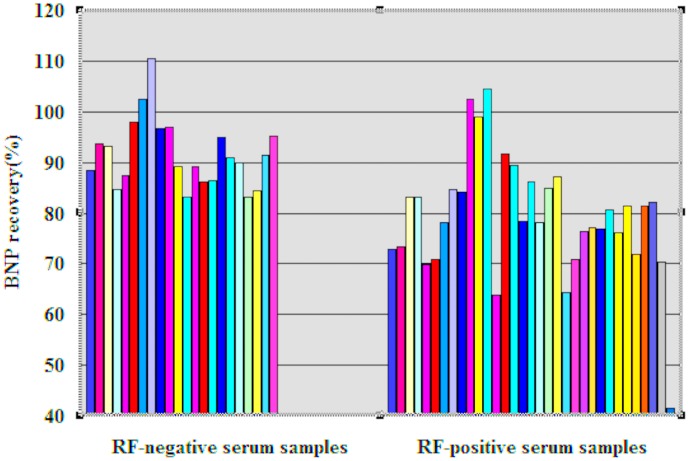
BNP recovery in RF-positive and RF-negative plasma samples.

BNP recovery was determined in 22 RF-negative (RF<20 IU/mL) and 33 RF-positive (RF levels ranged from 48 to 1420 IU/mL) plasma samples. The average recovery of BNP was 79.29% in the RF-positive plasma samples, with a maximum of 104.48% and a minimum of 41.62%, whereas that in the RF-negative plasma samples ranged from 83.03% to 110.48%, with a mean of 91.61% (see Figure 1 and Tables S1 and S2). Student's t test showed that the recovery of BNP was significantly lower in the RF-positive plasma samples than in the RF-negative plasma samples (t = 4.46, p<0.05). We therefore considered that normal BNP recovery fell within the range 79.19%-104.03% (two standard deviations from the mean value for the RF-negative plasma samples). BNP recovery in 17 of the 33 (51.52%) RF-positive plasma samples was lower than 79.19%, whereas only one sample had a BNP recovery of >104.03%.

### The effect of dilution on BNP recovery in RF-positive plasma samples

**Figure 2:**
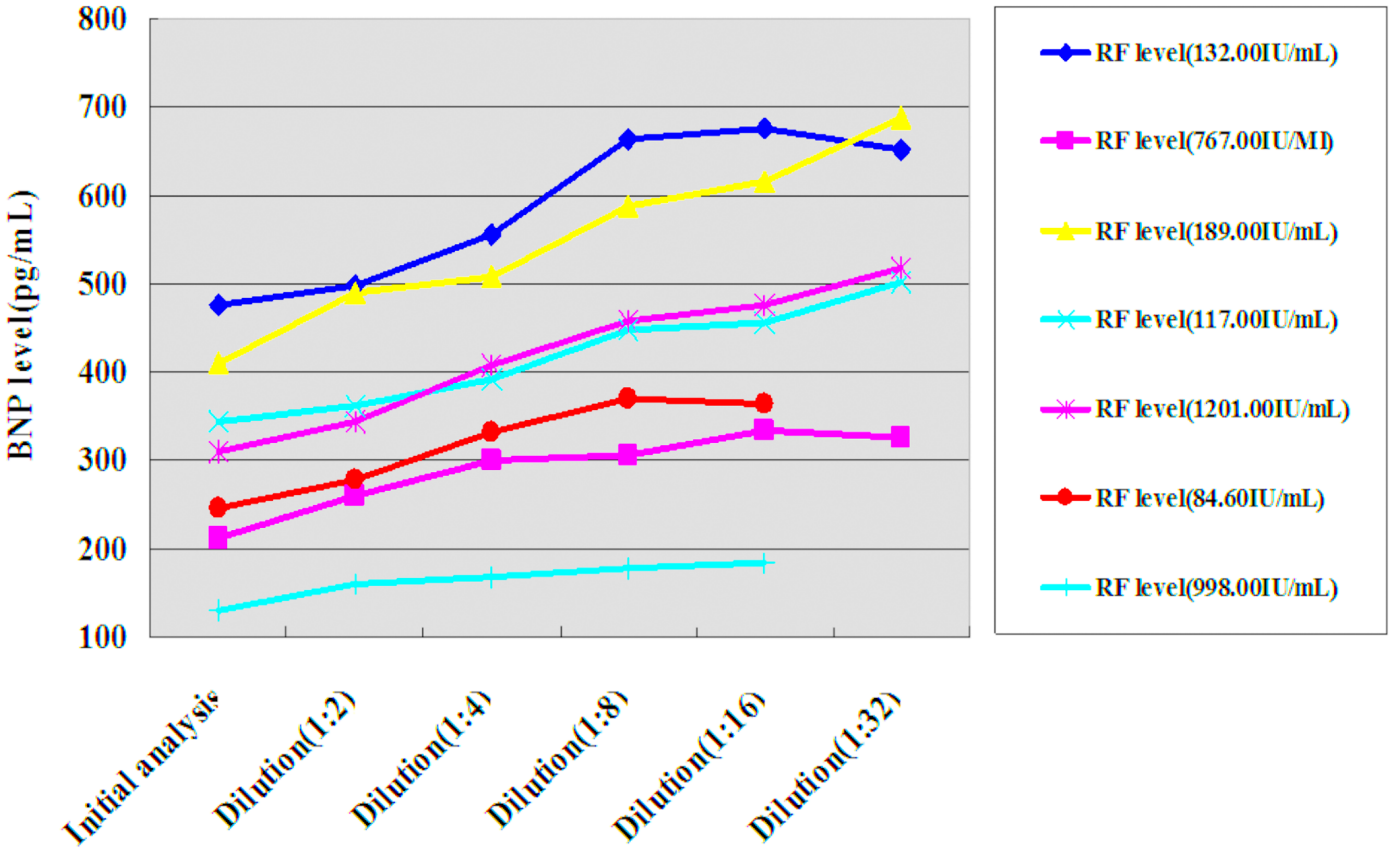
BNP levels measured in seven diluted RF-positive plasma samples.

**Figure 3:**
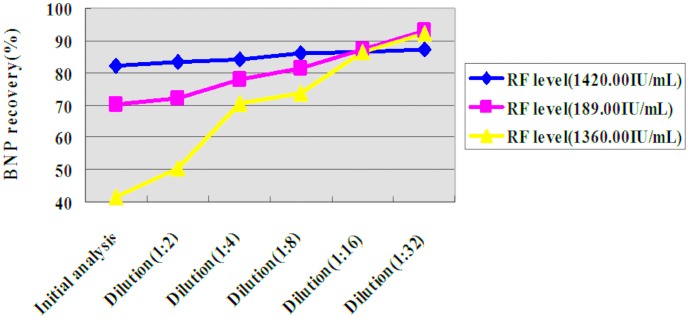
BNP recovery in three diluted RF-positive plasma samples.

Seven of the 33 RF-positive plasma samples were diluted, followed by quantitative determination of BNP levels. At a dilution of 1∶2, the BNP levels measured in three of the seven plasma samples were about 1.2 times their initial values. At a dilution of 1∶16, the average level of BNP measured in the seven samples was 1.48-fold the initial value, with a maximum of 1.58-fold and a minimum of 1.31-fold (see Figure 2 and Table 1). The ARCHITECT BNP assay was designed to have an upper 95% confidence interval imprecision of ≤12%. BNP levels measured in plasma samples that had discrepancies of ≥36% (3×12%) were considered to be significantly different. The measured BNP levels were significantly higher than the initial values in six of the samples at a dilution of 1∶16 (see Table 1). We also diluted three of the 33 plasma samples, for which the initial BNP recovery was 41.28%, 70.28% and 82.01%, respectively. At a dilution of 1∶16, the BNP recovery in all three of the samples was higher than 85% (see Figure 3 and Table 2).

**Table 1:** BNP levels measured in seven diluted RF-positive plasma samples.

No.	RF level (IU/mL)	Initial BNP level (pg/mL)	BNP level in diluted plasma samples (pg/mL)				
			1∶2	1∶4	1∶8	1∶16	1∶32
1	132.00	476.50	497.40	556.80	663.20	676.80	652.80
2	767.00	211.40	261.00	300.80	305.60	334.40	326.40
3	189.00	410.00	489.60	508.80	587.20	616.00	688.00
4	117.00	344.00	362.80	392.80	448.80	456.00	502.40
5	1201.00	309.30	344.80	407.60	457.60	476.80	518.40
6	84.60	245.10	278.20	332.80	370.40	364.80	U
7	998.00	130.90	160.00	168.00	177.60	184.00	U

BNP, B-type natriuretic peptide; RF, rheumatoid factor; U, undetected.

**Table 2:** BNP recovery in three diluted RF-positive plasma samples.

No.	RF level (IU/mL)	Added BNP (pg/mL)	BNP recovery in diluted samples					
			Initial	1∶2	1∶4	1∶8	1∶16	1∶32
1	1420.00	1214.80	82.01	83.36	84.01	86.11	86.46	87.09
2	189.00	2389.80	70.28	71.94	78.04	81.54	87.28	93.18
3	1360.00	1016.60	41.62	50.61	70.51	73.81	86.29	92.23

BNP, B-type natriuretic peptide; RF, rheumatoid factor.

### The effect of declined RF level on the measured BNP levels

**Figure 4:**
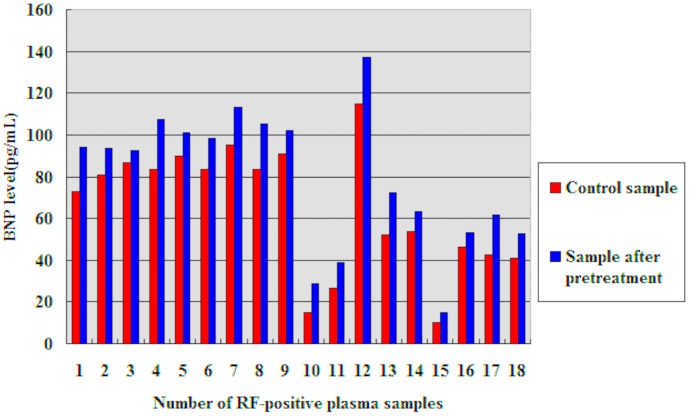
A comparison of the BNP levels measured in RF-positive plasma pretreated with or without IgG-sensitive latex particles.

Eighteen RF-positive plasma samples were pretreated with IgG-sensitive latex particles followed by quantitative determination of the BNP levels. Although the maximum decline rate of the RF levels was 50.58%, the BNP levels measured in the RF-positive samples increased significantly after pretreatment(t = 10.27, p = 0.00 <0.05) (see Figure 4 and Table 3).

**Table 3:** BNP levels measured in RF-positive plasma samples pretreated with IgG-sensitive latex particles.

No.	RF level (IU/mL)		BNP level (pg/mL)	
	Control sample	Pretreated sample	Control sample	Pretreated sample
1	30.51	16.70	73.00	93.97
2	85.80	47.51	80.90	93.33
3	70.23	53.31	86.80	92.48
4	139.00	68.70	83.40	107.46
5	498.00	327.00	90.00	101.21
6	620.00	512.00	83.70	98.59
7	368.00	244.00	95.40	113.25
8	846.00	625.00	83.30	105.29
9	530.00	408.00	90.90	101.93
10	15.10	9.94	15.00	28.80
11	549.00	338.00	26.50	38.90
12	429.00	270.00	115.00	137.00
13	610.00	520.00	51.90	72.10
14	214.00	190.00	53.70	63.00
15	782.00	735.00	10.00	14.90
16	516.00	385.00	46.50	53.30
17	23.00	10.50	42.60	61.60
18	25.10	13.20	40.80	52.50

BNP, B-type natriuretic peptide; IgG, immunoglobulin G; RF, rheumatoid factor.

### The correlation between the RF level and BNP recovery

Bivariate correlations tests of the 33 RF-positive samples showed that BNP recovery was not associated with the RF level when the plasma RF level ranged from 48 to 1420 IU/mL (r = −0.28, p = 0.12 >0.05). To exclude the influence of BNP heterogeneity on recovery, we added standard BNP (2614 pg/mL) to 14 of the RF-positive plasma samples, the RF levels of which ranged from 48 to 2720 IU/mL. The bivariate correlations tests also showed that BNP recovery did not correlate with the plasma RF concentration (r = −0.36, p = 0.20 >0.05). In addition, BNP recovery in two of the 14 RF-positive samples was higher than 104.03% (the mean value plus two standard deviations of the RF-negative samples).

## Discussion

A primary role of the clinical laboratory is to provide results that do not cause misdiagnosis of disease or mismanagement of illness. Unfortunately, this goal has not been completely achieved for immunoassays due to endogenous interferences [19]–[20]. Endogenous interfering substances in plasma, including human anti-mouse antibodies and heterophilic antibodies, can interfere with the immunoassay process and cause the inaccurate quantification of BNP. However, no study has reported that RF interferes with the BNP CMIA. We performed a recovery study by diluting high-concentration BNP with RF-positive and RF-negative plasma samples. In the RF-negative plasma samples, the average recovery of BNP was 91.61%, whereas it decreased to 79.29% in the RF-positive plasma samples; moreover, 17 of the 33 (51.52%) RF-positive plasma samples had a BNP recovery of <79.19%. Compared with the RF-negative plasma samples, the BNP recovery decreased significantly in the RF-positive plasma samples. These results suggested that potential endogenous interfering substances were present in the RF-negative plasma samples and that RF was a candidate endogenous interfering substance.

Plasma or serum samples suspected of containing interfering substances can be identified by three independent tests in clinical laboratory use: commercially available blocking reagents, serial dilutions, and repeated analyses using different immunoassay platforms [21]. In previous studies, we found that serial dilutions and the use of commercially available blocking reagents were two effective methods of identifying positive or negative interference from RF [6]–[7]. Here, seven RF-positive plasma samples were diluted 1∶2, 1∶4, 1∶8, 1∶16, and 1∶32, after which the BNP levels were measured. A 1∶2 dilution of three samples resulted in a ∼20% increase in the measured BNP level. At a dilution of 1∶16, the measured BNP level in six of the seven RF-positive plasma samples increased by >36%, which was three times the imprecision value provided by the manufacturer. In addition, we selected three samples with levels of recovery of BNP of 41.28%, 70.28%, and 82.01%, respectively, which represented severe, moderate, and mild declines in BNP recovery, respectively. At a dilution of 1∶16, the BNP recovery in all three of the samples was higher than 85%. These results showed that serial dilutions could significantly improve the recovery of BNP in RF-positive plasma samples, and therefore further supported the presence of endogenous interfering substances in the RF-positive plasma samples.

The use of commercially available blocking reagents is another method to identify interfering substances [6]–[7]. We used IgG-sensitive latex particles, which are generally used to determine the presence of RF in serum or plasma, to bind specifically to human RF followed by centrifugation at 13,000 rpm for 10 min. Although the RF was not removed completely from the supernatant using these particles, the measured BNP levels were significantly higher in the RF-positive plasma samples after pretreatment with IgG-sensitive latex particles. These data supported our suspicion that RF causes a negative interference in BNP CMIA. The probability of a negative interference rate from RF assuming a prevalence of heart failure of 80% in symptomatic patients was calculated to be 33% according to Bayes theorem [21]), a figure not dissimilar to ∼50% found in this study. Therefore, in order to determinate BNP level in RF-positive plasma samples with better accuracy, we suggest that samples need to be pretreated with commercially available blocking reagents such as heterophil-blocking reagent [17], [21].

As RF caused a negative interference in the BNP CMIA, we then analyzed whether the recovery of BNP was associated with the plasma RF level. When the RF concentration ranged from 48 to 1420 IU/mL, the recovery of BNP was not associated with the plasma RF concentration. To exclude any possible bias introduced by the diversity/variability of high-concentration BNP plasma, another recovery experiment was carried out by diluting a high-concentration standard BNP with RF-positive plasma at a ratio of 1∶9. When the RF level ranged from 48 to 2720 IU/mL, there was also no significant relationship between the BNP recovery and plasma RF concentration. Supporting this finding, our previous study showed that the decline in the rates of HBsAg signal/cut-off values was not associated with the serum RF concentration in an HBsAg ELISA [5]. Furthermore, our previous and present studies provided evidence that other important aspects such as binding affinity/avidity of RF affected the pattern and degree of interference in immunoassays [21]. It has been reported that RF leads to a false-positive or higher results in immunoassays [7]–[10]. Three of the 47 untreated RF-positive plasma samples had a BNP recovery of >104.03%, indicating that RF could also result in the false elevation of the measured BNP level.

In summary, this study showed that the susceptibility of the BNP CMIA to interference from RF led to predominantly (but not exclusively) lower results and, moreover, that the recovery of BNP was not significantly associated with the plasma RF concentration. Therefore, care may be needed in interpreting BNP results, particularly in patients with RA, in patients older than 60 years of age, and those with a high plasma RF concentration. When the RF in plasma is elevated, we highly recommend determining the plasma BNP level after pretreatment with commercially available blocking reagents prior to the initiation of denying patient's necessary treatment.

## Supporting Information

Table S1 **BNP recovery in RF-negative plasma samples.**
(DOC)

Table S2 **BNP recovery in RF-positive plasma samples.**
(DOC)
