# Supplementary material for: Negative Interference by Rheumatoid Factor of Plasma B-Type Natriuretic Peptide in Chemiluminescent Microparticle Immunoassays
Source: PLoS One. 2014 Aug 21;9(8):e105304. doi: 10.1371/journal.pone.0105304 (PMC4140727; doi:10.1371/journal.pone.0105304)
Supplement: Table S1 — BNP recovery in RF-negative plasma samples. (DOC) [file pone.0105304.s001.doc]

| No. | Basic levels  of BNP (pg/mL) | BNP added (pg/mL) | BNP recovery (%) |
| --- | --- | --- | --- |
| 1 | 50.6 | 1101.6 | 88.39 |
| 2 | 39.5 | 1101.6 | 93.79 |
| 3 | 30.5 | 1101.6 | 93.20 |
| 4 | 35.9 | 1101.6 | 84.59 |
| 5 | 29.9 | 1101.6 | 87.34 |
| 6 | 37.9 | 945.4 | 98.04 |
| 7 | 54.5 | 945.4 | 102.44 |
| 8 | 35.5 | 945.4 | 110.48 |
| 9 | 34 | 989.7 | 96.70 |
| 10 | 12.7 | 989.7 | 96.87 |
| 11 | 17.9 | 989.7 | 89.11 |
| 12 | 34.7 | 989.7 | 83.03 |
| 13 | 35.5 | 989.7 | 89.27 |
| 14 | 31.3 | 989.7 | 86.02 |
| 15 | 13.2 | 989.7 | 86.41 |
| 16 | 42.7 | 989.7 | 94.95 |
| 17 | 23.3 | 989.7 | 90.83 |
| 18 | 25.6 | 989.7 | 89.95 |
| 19 | 42.1 | 989.7 | 83.10 |
| 20 | 32 | 989.7 | 84.44 |
| 21 | 66.7 | 989.7 | 91.50 |
| 22 | 24.5 | 989.7 | 95.07 |
| Mean±SD |  |  | 91.61±12.42 |

BNP, B-type natriuretic peptide; RF, rheumatoid factor; SD, standard deviation
