# Supplementary material for: Negative Interference by Rheumatoid Factor of Plasma B-Type Natriuretic Peptide in Chemiluminescent Microparticle Immunoassays
Source: PLoS One. 2014 Aug 21;9(8):e105304. doi: 10.1371/journal.pone.0105304 (PMC4140727; doi:10.1371/journal.pone.0105304)
Supplement: Table S2 — BNP recovery in RF-positive plasma samples. (DOC) [file pone.0105304.s002.doc]

| No. | RF levels  (IU/mL) | Basic levels  of BNP (pg/mL) | BNP added  (pg/mL) | BNP recovery  (%) |
| --- | --- | --- | --- | --- |
| 1 | 285 | 25.6 | 945.4 | 72.84 |
| 2 | 767 | 20.6 | 945.4 | 73.37 |
| 3 | 1040 | 19.6 | 945.4 | 83.20 |
| 4 | 1420 | 16.3 | 945.4 | 83.17 |
| 5 | 132 | 17.3 | 945.4 | 69.74 |
| 6 | 189 | 73.5 | 945.4 | 70.82 |
| 7 | 271 | 23.8 | 945.4 | 78.15 |
| 8 | 117.4 | 86.8 | 945.4 | 84.70 |
| 9 | 126.8 | 22.9 | 945.4 | 84.19 |
| 10 | 161.2 | 54.5 | 945.4 | 102.55 |
| 11 | 23.3 | 31.5 | 945.4 | 98.95 |
| 12 | 60.7 | 14.8 | 945.4 | 104.48 |
| 13 | 84.6 | 232.4 | 1101.6 | 63.88 |
| 14 | 123 | 13.5 | 1101.6 | 91.73 |
| 15 | 179 | 45.8 | 1101.6 | 89.49 |
| 16 | 180 | 14.5 | 1101.6 | 78.35 |
| 17 | 197 | 18.8 | 1101.6 | 86.25 |
| 18 | 442 | 12.9 | 1101.6 | 78.00 |
| 19 | 498 | 12.3 | 1101.6 | 84.92 |
| 20 | 998 | 63.7 | 1101.6 | 87.03 |
| 21 | 846 | 50.3 | 887.8 | 64.32 |
| 22 | 498 | 10 | 887.8 | 70.96 |
| 23 | 85.8 | 13 | 887.8 | 76.48 |
| 24 | 53.3 | 18.3 | 887.8 | 77.16 |
| 25 | 181 | 15.2 | 887.8 | 76.82 |
| 26 | 1180 | 18.5 | 887.8 | 80.54 |
| 27 | 767 | 16.2 | 887.8 | 76.03 |
| 28 | 620 | 23.1 | 887.8 | 81.44 |
| 29 | 48 | 19.6 | 887.8 | 71.75 |
| 30 | 285 | 18.7 | 887.8 | 81.32 |
| 31 | 1420 | 16.3 | 1214.8 | 82.01 |
| 32 | 189 | 33.5 | 2389.8 | 70.28 |
| 33 | 1360 | 12.10 | 1016.6 | 41.62 |
| Mean±SD |  |  |  | 79.29±23.50 |

BNP, B-type natriuretic peptide; RF, rheumatoid factor; SD, standard deviation
